# Quantification of biological range uncertainties in patients treated at the Krakow proton therapy centre

**DOI:** 10.1186/s13014-022-02022-5

**Published:** 2022-03-09

**Authors:** Magdalena Garbacz, Jan Gajewski, Marco Durante, Kamil Kisielewicz, Nils Krah, Renata Kopeć, Paweł Olko, Vincenzo Patera, Ilaria Rinaldi, Marzena Rydygier, Angelo Schiavi, Emanuele Scifoni, Tomasz Skóra, Agata Skrzypek, Francesco Tommasino, Antoni Rucinski

**Affiliations:** 1 Institute of Nuclear Physics Polish Academy of Sciences, 31342 Kraków, Poland; 2 GSI Helmholtzzentrum fur Schwerionenforschung, 64291 Darmstadt, Germany; 3 The Technical University of Darmstadt, 64289 Darmstadt, Germany; 4 National Oncology Institute, National Research Institute, Krakow Branch, 31115 Kraków, Poland; 5 University of Lyon, CREATIS, CNRS UMR5220, Inserm U1044, INSA-Lyon, Université Lyon 1, Centre Léon Bérard, France; 6 INFN - Section of Rome, 00185 Rome, Italy; 7 Department of Basic and Applied Sciences for Engineering, Sapienza University of Rome, 00161 Rome, Italy; 8 ZonPTC/Maastro Clinic, Maastricht, The Netherlands; 9 Trento Institute for Fundamental Physics and Applications, TIFPA-INFN, 38123 Povo, Trento, Italy; 10 Awesome Industries Solutions, 31026 Kraków, Poland; 11 Department of Physics, University of Trento, 38123 Povo, Trento, Italy; 12 University of Lyon, Université Claude Bernard Lyon 1, CNRS/IN2P3, IP2I Lyon, UMR 5822, Villeurbanne, France

**Keywords:** Biological range extension, Monte Carlo, Proton therapy, Range uncertainties, Variable RBE

## Abstract

**Background:**

Variable relative biological effectiveness (vRBE) in proton therapy might significantly modify the prediction of RBE-weighted dose delivered to a patient during proton therapy. In this study we will present a method to quantify the biological range extension of the proton beam, which results from the application of vRBE approach in RBE-weighted dose calculation.

**Methods and materials:**

The treatment plans of 95 patients (brain and skull base patients) were used for RBE-weighted dose calculation with constant and the McNamara RBE model. For this purpose the Monte Carlo tool FRED was used. The RBE-weighted dose distributions were analysed using indices from dose-volume histograms. We used the volumes receiving at least 95% of the prescribed dose (V95) to estimate the biological range extension resulting from vRBE approach.

**Results:**

The vRBE model shows higher median value of relative deposited dose and D95 in the planning target volume by around 1% for brain patients and 4% for skull base patients. The maximum doses in organs at risk calculated with vRBE was up to 14 Gy above dose limit. The mean biological range extension was greater than 0.4 cm.

**Discussion:**

Our method of estimation of biological range extension is insensitive for dose inhomogeneities and can be easily used for different proton plans with intensity-modulated proton therapy (IMPT) optimization. Using volumes instead of dose profiles, which is the common method, is more universal. However it was tested only for IMPT plans on fields arranged around the tumor area.

**Conclusions:**

Adopting a vRBE model results in an increase in dose and an extension of the beam range, which is especially disadvantageous in cancers close to organs at risk. Our results support the need to re-optimization of proton treatment plans when considering vRBE.

**Supplementary Information:**

The online version contains supplementary material available at 10.1186/s13014-022-02022-5.

## Background

Proton therapy (PT) is an effective technique for treating specific types of cancer, such as head and neck tumors [1]. Thanks to the inverse depth-dose profile of protons with respect to photons and finite proton beam range, proton radiation therapy gives the possibility to efficiently treat malignant tumors, administering lower doses to healthy tissues compared to photon radiation therapy [2]. Proton range for dose distribution is usually defined as the depth where the dose drops to 80% or 90% of planned dose according to Paganetti [3]. Proton radiation therapy gives the possibility to efficiently treat malignant tumors, administering lower doses to healthy tissues compared to photon radiation therapy [2]. Protons also exhibit an increased relative biological effectiveness (RBE, defined as the ratio of photon to proton physical dose needed to achieve the same biological effect), which makes them more effective than photons [4]. In clinical practice, safety margins are added to the clinical target volume (CTV), thus generating the planning target volume (PTV) to ensure that the entire potentially malignant tissue is irradiated by taking into account the range uncertainty and tumor motion. The conformity of PT dose distributions, that is essential for patient safety, is obtained by the combination of proton beam properties, application of multiple treatment fields and intensity modulation technique. Nevertheless, robustness of PT treatment plans is challenged by proton beam range uncertainty in the patient [5].

An emerging issue of PT treatment planning is the variation of RBE values across the target volume; currently, it is assumed that RBE has a constant value of 1.1 in clinical practice. In fact, radiobiological experiments show that the RBE values at the end of the proton range (i.e. Bragg peak distal fall-off) increases up to 2 [6], according to the review in Ilicic et al. [7]. The clinical evidence for an enhanced proton RBE at the distal edges has been reported in the very recent summary report of an EPTN workshop [8]. This variation of RBE along the proton path is an additional source of uncertainty in PT treatment planning. In order to obtain more precise information on dose distribution in patients, advanced dose calculation methods based on analytical or Monte Carlo (MC) algorithms are used for treatment planning (e.g. Eclipse [9], RayStation [10]) or to support treatment planning, quality assurance and research (e.g., TOPAS [11], GATE [12], Fluka [13], FRED [14], gPMC [15], MCsquare [16]). These tools can calculate physical and RBE-weighted dose (D_RBE_) distributions. The latter is typically obtained, for protons, with a parametric model using the dose-averaged linear energy transfer (LET_d_) [4] and the information on radiosensitivity of different tissue types characterized by the α/β ratio [17, 18]. Identification of the increased LET_d_ regions (which also increase the RBE values) during treatment planning might be used to minimize the LET_d_ and RBE outside the PTV, especially in organs at risk (OARs) [19, 20].

In this paper we investigate, quantify and compare biological uncertainties of clinical PT treatment plans with constant RBE (cRBE) and variable RBE (vRBE), focusing on the range uncertainty and evaluating clinical parameters of dose-volume histograms (DVHs). We performed MC simulations of clinical treatment plans using FRED (Fast paRticle thErapy Dose evaluator) [14]. We used simulation data to propose a method of quantification of biological range extension (or biologically effective range, as defined in Grün et al. [21]). Further, we compared biological uncertainties of the treatment plan with uncertainties resulting from its robustness to patient positioning and computed tomography (CT) calibration.

## Methods and materials

### Patients database description

In our study we used CT images (Siemens Somatom Definition AS), as well as treatment plans and dose distributions calculated in the treatment planning system (TPS) Eclipse 13.6 for 95 patients with brain and skull base tumors, treated with protons from November 2016 to September 2018 at the Cyclotron Centre Bronowice (CCB) in Krakow. The patients underwent intensity-modulated proton therapy (IMPT) with fraction and total RBE-weighted doses (accounting for RBE = 1.1) ranging from 1.8 to 2.0 Gy(RBE) and 36 to 74 Gy(RBE), respectively. The treatments were prepared with multi-field optimization (MFO) and carried out in 1–3 stages, where stage 2 and 3 were the boost plans. The patients were divided according to the tumor type into two groups, i.e., brain patients and skull base patients, which is presented in Table 1. Information on the PTV volume, prescribed dose and diagnosis for each patient can be found in Table S1 in Additional file 1. Aiming at the coherence of the patient database and most clinically representative results, we excluded 20 patients from the database with tumor localization other than brain or head and neck (H&N), pediatric cases, replanned patients and treatment plans optimized with single field uniform dose (SFUD) approach.

**Table 1:** Patient database grouped according to tumor type

Group 1: Brain patients (1–50)			Group 2: Skull base patients (51–95)		
Number of patients	1 stage	50	Number of patients	1 stage	14
				2 stages	28
	Sum	50		3 stages	3
				Sum	45
Kind of tumor	Gliomas	50	Kind of tumor	Chordomas	28
				Chondrosarcomas	17
Prescribed dose	Max	61.2 Gy(RBE)	Prescribed dose	Max	74.0 Gy(RBE)
	Min	36.0 Gy(RBE)		Min	70.0 Gy(RBE)
	Median	54.0 Gy(RBE)		Median	74.0 Gy(RBE)
Number of ﻿field﻿s	Max	4	Number of fields	Max	6
	Min	2		Min	2
	Median	3		Median	4
α/β PTV	6 Gy		α/β PTV	4 Gy	

### LET, RBE and dose calculations in FRED MC

In this work we performed MC calculations of D_RBE_ and LET_d_ distributions delivered by treatment plans prepared in TPS. The MC calculations were made in a patient anatomy defined by its CT images, resampled from the original grid size of 0.67 × 0.67 × 1.2 mm^3^ to 1.5 × 1.5 × 1.5 mm^3^. The calculations were performed with the MC tool FRED. FRED allows for particle tracking on graphical processing units (GPUs), which shortens the calculation time for the whole treatment plan to several minutes, which is about 1000 times faster than general purpose MC tools executed on central processing units (CPUs) [14]. The beam model used for patient treatment at the Krakow PT center was implemented in FRED and validated against commissioning data, measurements in homogeneous and heterogeneous media and standard clinical calculations [22]. FRED includes several radiobiological models, both parametric and table based (e.g. Carabe [23], McNamara [24], LEM1 [25]), allowing storing RBE distributions voxel by voxel.

In this paper we computed D_RBE_ distributions using the parametric vRBE model by McNamara since it is based on the most recent and comprehensive set of radiobiological data and accounts for the LET_d_ distribution in considered volume [24]. The α/β ratios were chosen based on the tumor type and overview presented in van Leeuwen et al. [26]. We used α/β ratios of 6 Gy and 4 Gy for brain patients and skull base patients, respectively, while D_RBE_ to normal tissue was computed using α/β of 2 Gy. RBE values were extracted for each patient individually for the voxels with the RBE-weighted dose values exceeding 5% of the prescribed dose. We studied the differences in RBE values in the PTV and OARs, where elevated RBE can cause post-treatment side effects in patients. The LET_d_ values were calculated in each voxel from all primary and secondary protons as a sum of deposited energy of all events weighted by the particle stopping power as in Polster et al. [27]:

1 $${\text{LET}}_{{\text{d}}} = \, 1/\rho \cdot \Sigma [{\text{dE}} \cdot ({\text{dE/dx}})]/\Sigma {\text{dE}},$$

where *dE*—deposited energy, *dx*—path length, *ρ*—density of the medium and Σ*dE*—the sum runs on all events of energy deposition.

### Evaluation of RBE-weighted dose

To compare the D_RBE_ distributions computed with cRBE and vRBE, DVHs and the DVH parameters were computed for each patient and each dose distribution. The mean and maximum doses in PTV as well as in brainstem and chiasm were evaluated. The DVHs were generated using the dicompyler-core library [28]. All data analysis was performed using in-house Python scripts that were validated against Eclipse TPS.

### Biological range extension

**Fig. 1:**
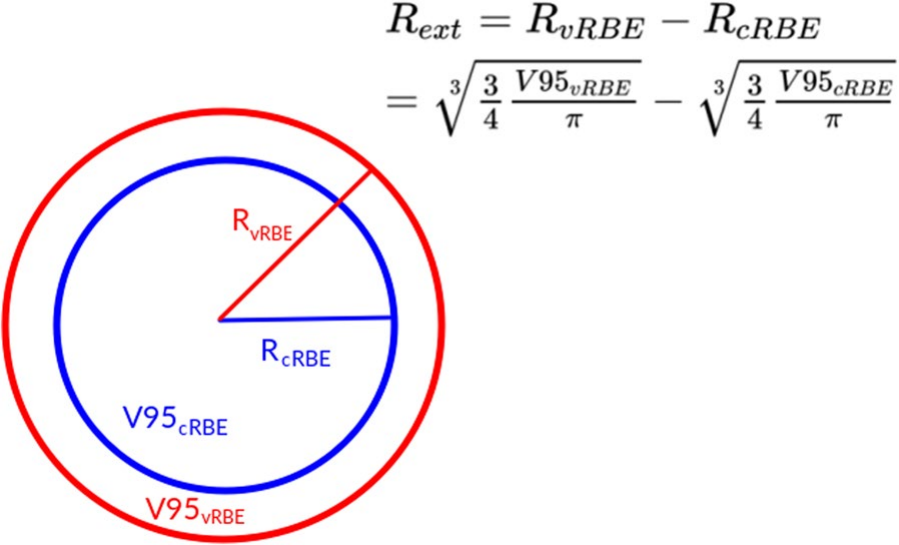
Schematic presentation of biological range extension estimation method

Most of the patient plans consist of 3–4 treatment fields from different directions, which were arranged around the target, therefore the range can be extended in different directions. For evaluation of biological range extension we used an approximation, by assuming that the volume covered with at least 95% of the prescribed dose (V95) computed with cRBE and vRBE has a spherical shape. We thus defined biological range extension as the difference between radiuses:

2 $${\text{R}}_{{{\text{ext}}}} = {\text{ R}}_{{{\text{vRBE}}}} - {\text{R}}_{{{\text{cRBE}}}} ,$$

where R_vRBE_—radius of sphere of V95 volume computed with vRBE, R_cRBE_—radius of sphere of V95 volume computed with cRBE. The schematic representation of this method is presented in Fig. 1.

### Comparison of D_RBE_ uncertainty and treatment plan robustness

We evaluated physical and biological uncertainty of treatment plans by comparing the DVHs of D_RBE_ distributions for PTV and OAR structures computed with: cRBE versus vRBE, and cRBE versus treatment plan robustness uncertainty bands. We have chosen the most critical structures (i.e. OARs with the highest dose) for tumor types selected for this study. We also analyzed the dose in other OAR including the spinal cord, which in majority of the cases is substantially below the dose constraint for this organ regardless of the RBE modeling approach. The robustness analysis was performed in FRED following the clinical procedure implemented at CCB: each clinical treatment plan was recalculated with cRBE for 12 cases: modifying CT image Hounsfield units (HU) values by ± 3.5% and translating the treatment plan isocenter of ± 2 mm in X, Y and Z directions. The DVHs with error bands were generated for PTV and OARs. Generally, the PTV was created by expanding the CTV with a safety margin of 3 mm, taking into account patient anatomy variations and patient positioning. In addition, CTV was extended to a complementary technical PTV structure (PTV_tech_) that includes the CT calibration curve uncertainty and is equivalent to a safety margin of 3.5% of the maximal beam range plus 1 mm for a given treatment field. The treatment dose was eventually prescribed to the PTV_tech_ that is the envelope of the maximum safety margin of PTV or PTV_tech_. The aim was to perform a comparative study of the dose variations originating from: the vRBE modelling against the CT translation, which mimics the patient mispositioning and the uncertainty of HU values due to CT scanner calibration, both performed with cRBE.

To quantify the differences between DVHs for cRBE and vRBE models the DVH indices such as D_max_ (the maximum local dose in he considered structure), D_mean_ (the mean dose in the considered structure), D05 (the maximum dose covering 5% of the considered structure) and D95 (the maximum dose covering 95% of the considered structure). The D95 and D05 parameters were analyzed for CTV and OARs, respectively, according to the formulas:

3 $${\text{CTV}}_{{{\text{diffRobust}}}} = {\text{D}}95_{{{\text{cRBE}}}} - \, \min \left( {{\text{D}}95} \right)_{{{\text{robust}}}} ,$$

4 $${\text{CTV}}_{{{\text{diffRBE}}}} = {\text{ D}}95_{{{\text{vRBE}}}} - {\text{D}}95_{{{\text{cRBE}}}} ,$$

5 $${\text{OAR}}_{{{\text{diffRobust}}}} = \, \max \left( {{\text{D}}05} \right)_{{{\text{robust}}}} - {\text{D}}05_{{{\text{cRBE}}}} ,$$

6 $${\text{OAR}}_{{{\text{diffRBE}}}} = {\text{D}}05_{{{\text{vRBE}}}} - {\text{D}}05_{{{\text{cRBE}}}} .$$

### Time performance

The time performance of FRED calculations was discussed before [14, 22]. In this study we simulated 10^4^ primary protons per pencil beam for each treatment plan. The mean(std) simulation time for brain patients was 2.75(1.35) min and for skull base patients 1.74(0.94) min, which makes FRED calculations sufficiently fast for its application in the clinical routine to support treatment planning and quality assurance.

### Statistical analysis

No a priori assumption on the distribution of the dose values was done. The 95% confidence intervals (2.5–97.5 percentile, CI_95%_) were calculated with the bootstrap method (100,000 iterations) around the median value for relative D_RBE_ values and biological range extension. Estimation of statistical difference between doses and changes in proton range using cRBE and vRBE was performed using the two-sided Wilcoxon signed-rank test [29].

## Results

### Validation of LET_d_ and D_RBE_ calculations in FRED

**Fig. 2:**
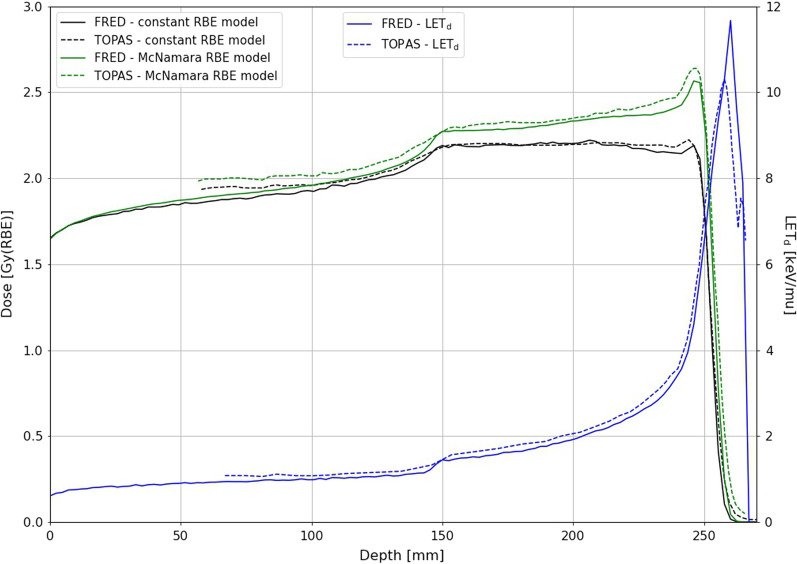
Comparison of D_RBE_ and LET_d_ calculations between FRED (solid lines) and TOPAS (dashed lines)

The accuracy of D_RBE_ and LET_d_ distribution calculations in FRED were validated against TOPAS simulations from McNamara et al. [30], which is presented on Fig. 2. The FRED simulation of a dose cube optimized with Eclipse TPS of modulation width from 150 to 250 mm was performed in a virtual water phantom with statistics of 10^4^ primary protons per pencil beam.

The maximum difference in D_RBE_ computed with the vRBE model in FRED and TOPAS (adapted from [30]) was 3%. The difference between maximum LET_d_ values was up to 13%, which does not substantially influence the RBE values in patients.

### Comparison of D_RBE_ in PTV

**Fig. 3:**
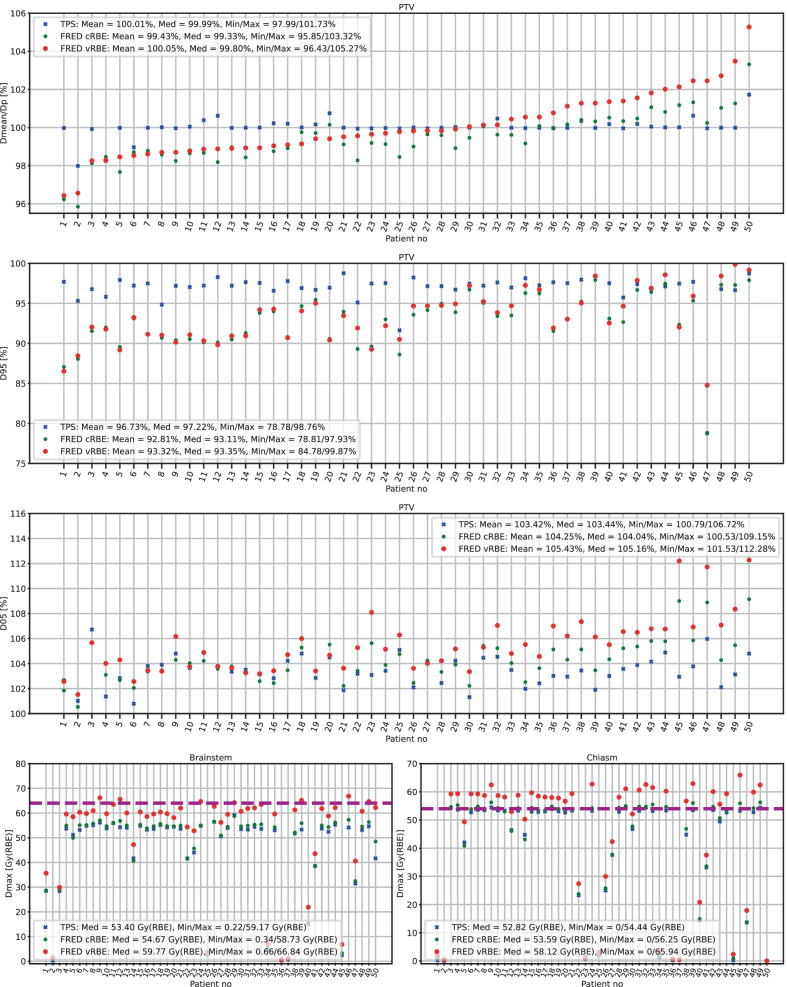
Brain patient dose analysis. (first panel) Relative mean doses (D_mean_) in PTV to prescribed dose (D_p_), (second panel) D95 in PTV, (third panel) D05 in PTV, (fourth and fifth panels) maximum doses (D_max_) in brainstem (left) and chiasm (right). Purple lines show dose constraints for brainstem surface (dashed line), brainstem core (dotted line) and chiasm (dashed line)

**Fig. 4:**
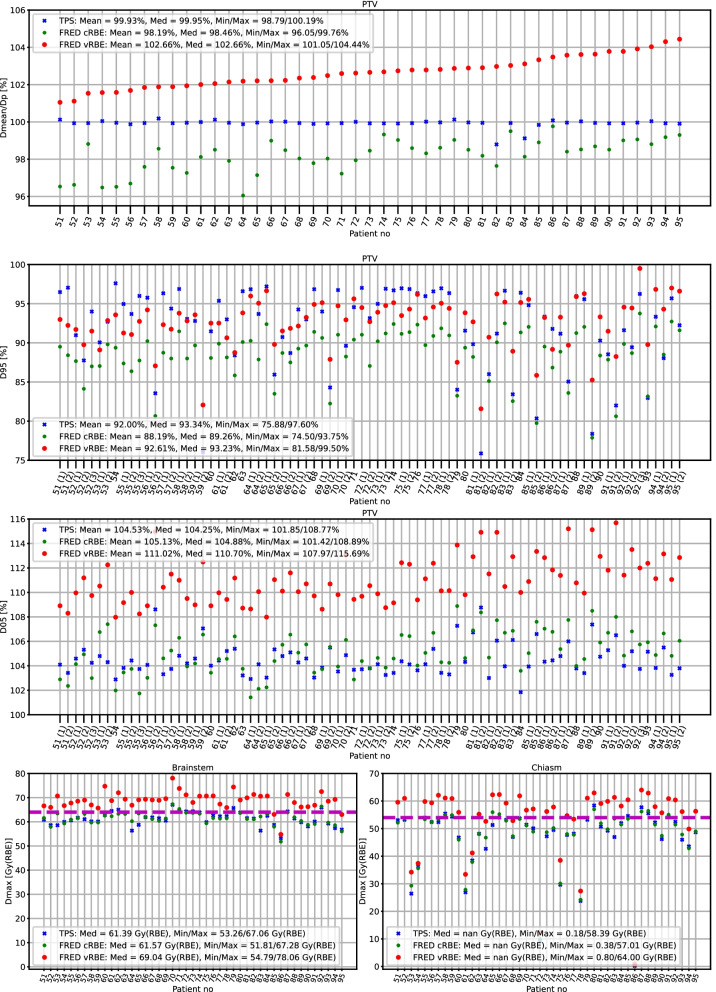
Skull base patient dose analysis. (first panel) Relative mean doses (D_mean_) in PTV to prescribed dose (D_p_), (second panel) D95 in PTV, (third panel) D05 in PTV, (fourth and fifth panels) maximum doses (D_max_) in brainstem (left) and chiasm (right). Purple lines show dose constraints for brainstem surface (dashed line), brainstem core (dotted line) and chiasm (dashed line)

Two patient groups: brain patients (Patient 1–50) and skull base patients (Patient 51–95) were analysed separately. Patients were ranked according to increasing mean vRBE-weighted dose in the PTV. The comparison of relative mean dose in PTV to the prescribed dose are presented on the top panels of Figs. 3 and 4, with mean, median, minimum and maximum doses over all patients provided in the legend. For brain patients, the CI_95%_ of D_mean_/D_p_ median was:

- [98.93, 99.83]% (median 99.33%) for FRED cRBE model and
- [99.28, 100.29]% (median 99.80%) for FRED vRBE model.

For skull base patients the CI_95%_ of D_mean_/D_p_ of median was

- [98.04, 98.56]% (median 98.44%) for FRED cRBE model and
- [102.23, 102.87]% (median 102.61%) for FRED vRBE model.

The *p* value from Wilcoxon test performed between D_RBE_ computed in FRED with cRBE and vRBE models was *p* < 0.05 (*p* = 1.8 × 10^–7^ and *p* = 5.2 × 10^–9^ for brain and skull base patients, respectively), which means that the mean deposited dose in the PTV relative to prescribed doses in PTV (D_mean_/D_p_) are significantly different.

The values of D95 and D05 parameters computed for both groups of patients are presented in Figs. 3 and 4. For the brain patients the D95 values significantly differ (*p* = 0.002) as well as D05 values (*p* = 2.1 × 10^–8^), between D_RBE_ distributions computed with the cRBE and vRBE models with CI_95%_ [91.54, 93.96] and [91.93, 94.67]. In the case of skull base patients the difference was also significant in both D95 (*p* = 1.9 × 10^–10^, CI_95%_ [88.20, 89.85] and [92.52, 93.82] for cRBE and vRBE models) and D05 (*p* = 1.1 × 10^–14^, CI_95%_ [104.56, 105.75] and [110.06, 111.19] for cRBE and vRBE models).

### Comparison of D_RBE_ in OARs

The maximum dose (D_max_) in brainstem and chiasm was analyzed for both groups of patients and presented on the fourth and the fifth panel of Figs. 3 and 4, respectively. The D_max_ in OARs was compared to the clinical constraints, which is 54 Gy(RBE) for brainstem core, 64 Gy(RBE) for brainstem surface and 54 Gy(RBE) for chiasm. The DVH analysis was performed on the whole brainstem structure. The median dose values computed with TPS and FRED for cRBE model are close to the dose constraints, while for the vRBE model the doses were much higher and often above dose constraints (up to 14 Gy above the limit), especially for skull base patients (73% and 100% of the patients for chiasm and brainstem, respectively).

### RBE distribution for vRBE model

In the PTV for brain patients (α/β = 6 Gy), the mean of the RBE values was 1.11, ranging from 1.07 to 1.31, whereas for skull base patients (α/β = 4 Gy) the mean RBE was 1.15 ranging from 1.09 to 1.49. In the brainstem the RBE values reached up to 2.71 and 2.36, while in chiasm the values were up to 2.16 and 2.19 for brain and skull base patients, respectively. The escalation of RBE values after the Bragg peak around 2.5 was also reported recently in Missiaggia et al. [31]. Note that depending on the local dose in a voxel, the high LET_d_ or RBE value may have severe or negligible impact on the OARs side effects. However, we found that for the majority of investigated patient cases, the RBE-weighted dose with vRBE has a median value of over 50 Gy(RBE), so even the slightly increased RBE can potentially impact the clinically relevant biological dose in brainstem or chiasm (with dose constraints for brainstem core and chiasm of 54 Gy(RBE)), and eventually have severe impact on these organs.

**Fig. 5:**
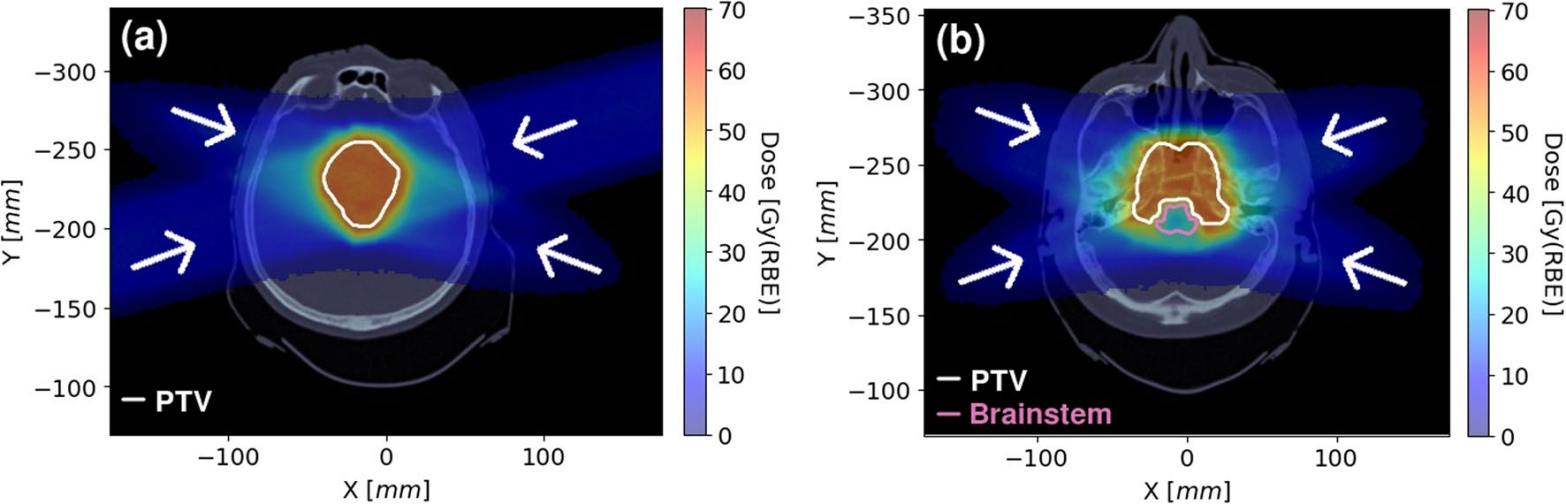
Dose distribution calculated with FRED using cRBE model. The treatment field directions are indicated by white arrows for Patient 49 (**a**) (gantry angles: 75°, 255°, 285°, 110°) and Patient 94 (1) (**b**) (gantry angles: 110°, 70°, 290°, 250°)

**Fig. 6:**
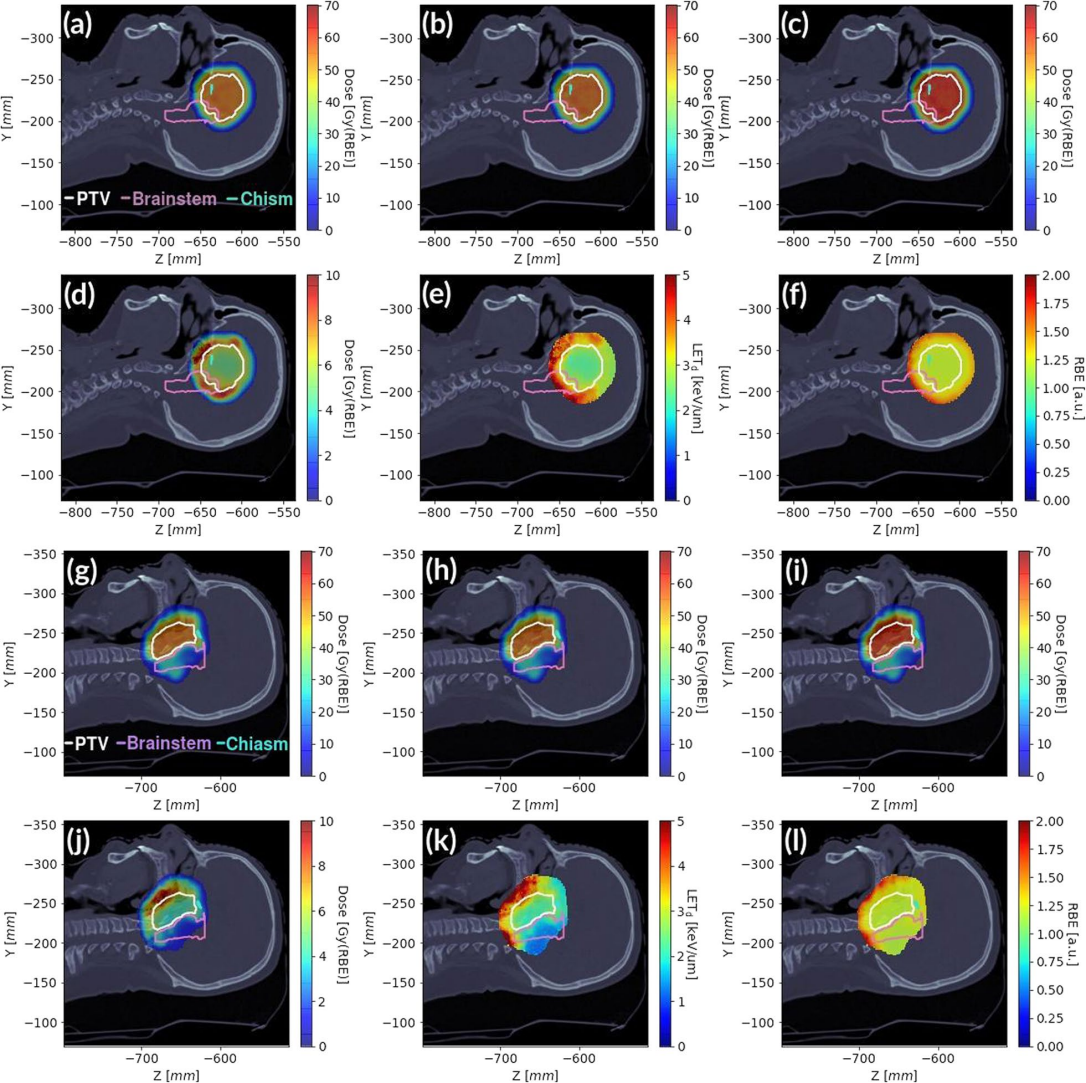
Examplonary distributions for brain (two upper rows, Patient 49) and skull base patient (two lower rows, Patient 94 (1)). **a** Dose TPS, **b** dose FRED cRBE, **c** dose FRED vRBE, **d** dose difference (vRBE − cRBE), **e** LET_d_, **f** RBE values (McNamara model), **g** dose TPS, **h** dose FRED cRBE, **i** dose FRED vRBE, **j** dose difference (vRBE − cRBE), **k** LET_d_, **l** RBE values (McNamara model) with contoured PTV (white), brainstem (pink) and chiasm (cyan)

The beam arrangement with the D_RBE_ calculated with FRED cRBE model is presented on Fig. 5. Figure 6 shows the D_RBE_, LET_d_ and RBE distributions with delineated PTV and OARs for an exemplary brain patient (panels (a)–(f) on Fig. 6) and skull base patient (panels (g)–(l) on Fig. 6). The dose difference between FRED cRBE and vRBE calculations is visible for both patient cases around the PTV contour. The increase of D_RBE_ computed using vRBE is the consequence of an increased RBE, especially at the distal edge of the dose fields.

### Mean biological range extension

The V95 volumes for brain patients computed with cRBE ranged from 59.75 to 1066.97 cm^3^ and with vRBE model from 100.43 to 1286.71 cm^3^. Due to application of vRBE for dose calculation, the mean(std) volume of V95 increased by 118.86(60.15) cm^3^. For skull base patients the V95 ranged from 8.30 to 575.49 cm^3^ for cRBE and from 19.82 to 727.17 cm^3^ for the vRBE model. The mean V95 volume increase (V95_diff_ = V95_vRBE_ − V95_cRBE_) was V95_diff_ = 63.31(33.98) cm^3^. The Pearson's test between V95 volume and biological range extension does not show any correlation for both groups of patients. It means that the size of irradiated volume does not determine the extent of the biologically effective range.

The mean R_cRBE_ for brain was 4.26 cm with CI_95%_ [4.02, 4.51] and R_vRBE_ was 4.70 cm with CI_95%_ [4.45, 4.96], whereas for skull base patients R_cRBE_ was 3.00 cm with CI_95%_ [2.84, 3.16] and R_vRBE_ 3.45 cm with CI_95%_ [3.29, 3.62]. Results for all patients correlated with the volume of the V95 are presented on Fig. 6. The mean biological range extension R_ext_ (formula(2)) for brain patients was R_ext_(brain) = 0.44(0.14) cm and for skull base patients R_ext_(skull base) = 0.45(0.11) cm.

**Fig. 7:**
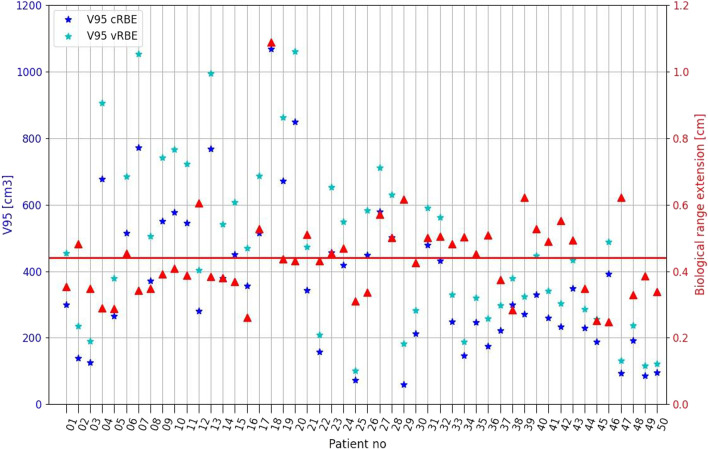
The V95 volumes and calculated biological range extension for brain patients. The solid line represents the mean range extension

**Fig. 8:**
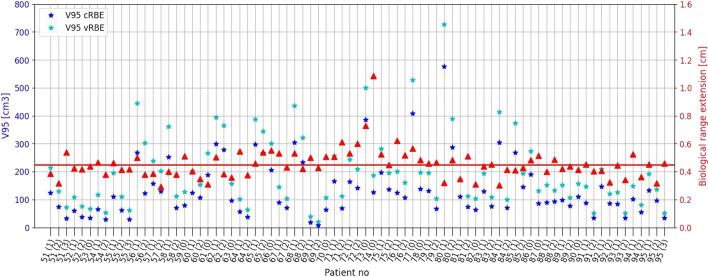
The V95 volumes and calculated biological range extension for skull base patients. The solid line represents the mean range extension

Results for all patients correlated with the volume of the V95 are presented on Figs. 7 and 8. The Pearson correlation coefficient between the V95 (calculated with vRBE) and biological range extension was 0.25 and 0.11 for brain and skull base patients, respectively. The results show that, at least for the patients considered in this study, the biological range extension does not significantly vary with the tumor localization and type.

### Comparison of D_RBE_ uncertainty and treatment plan robustness

**Fig. 9:**
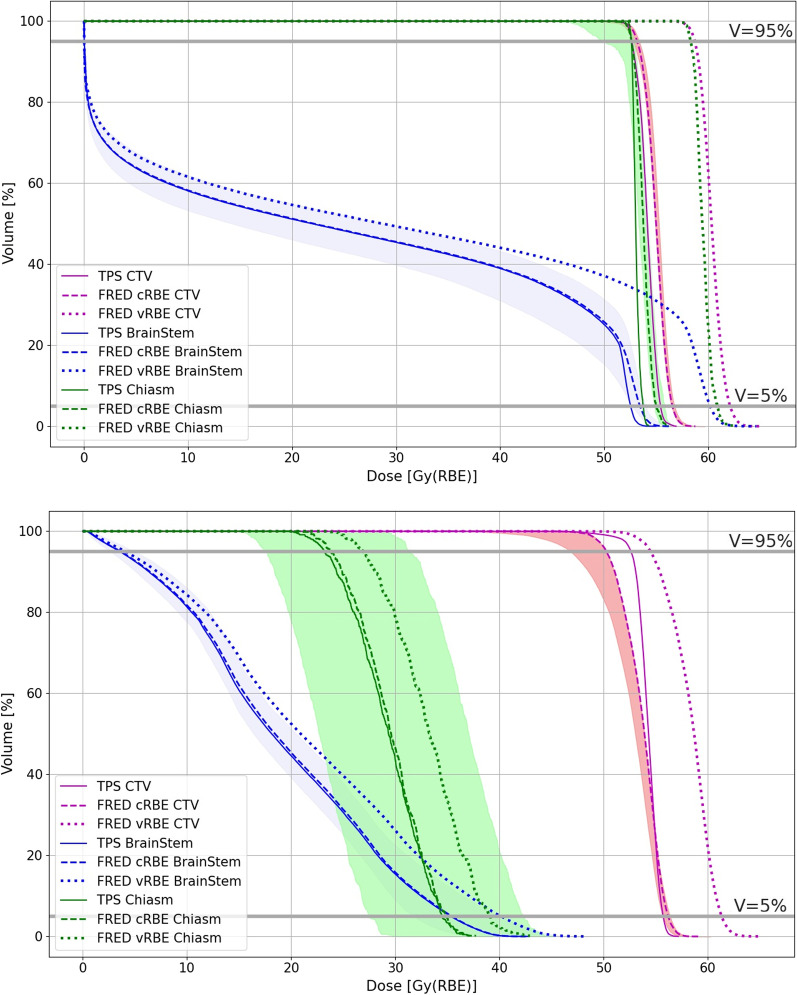
Robustness analysis around the FRED cRBE model (shaded regions) for brain patient (Patient 49, upper panel) and skull base patient (Patient 94 (1), lower panel)

The results were analyzed and presented as DVHs of CTV (magenta curves), chiasm (green curves) and brainstem (blue curves) volume in Fig. 9. The shaded regions around the DVH computed for the dose calculation performed with cRBE model show the variation of the dose distribution resulting from the robustness analysis. The mean difference for CTV from D95 parameter (calculated by formulas (3) and (4)) were:

- CTV_diffRobust_ was 0.7(1.0) Gy(RBE) and 3.2(2.6) Gy(RBE),
- CTV_diffRBE_ was 3.9(0.7) Gy(RBE) and 3.4(1.8) Gy(RBE)

for brain and skull base patients, respectively. The mean differences for OARs form D05 parameter (calculated by formulas (5) and (6)) were:

iii. OAR(brainstem)_difRobust_: 1.4(2.0) and 2.6(1.6) Gy(RBE), while OAR(chiasm)_difRobust_: 1.7(2.4) and 2.3(3.5) Gy(RBE),
iv. OAR(brainstem)_difRBE_: 4.5(2.3) and 3.2(1.9) (RBE), while OAR(chiasm)_difRBE_: 4.3(2.3) and 3.4(2.3) Gy(RBE)

for brain and skull base patients, respectively.

Results for the selected brain and skull base patients show that incorporating the vRBE model can modify the shape of the DVH (due the dose escalation) compared to the cRBE model (as also reported in Tommasino et al. [32]), to a level comparable or even higher than changes caused by patient misalignment or CT number uncertainty (shaded areas), especially in high dose regions.

## Discussion

The brain and skull base tumors are particularly challenging targets, because they are often localized very close to the critical structures (e.g. brainstem or chiasm, see Fig. 5) or even overlap with them. Our results show that the application of vRBE model causes an increase of the dose in PTV and OARs (see Fig. 3 and 4), which in turn enlarges the region covered by the high D_RBE_. For brain and H&N patients Yepes et al. [33] showed RBE values greater than 1.4 and 1.3, respectively using α/β = 2 Gy, which is a similar result to one obtained in this study. We have shown that for glioma tumor tissues with high α/β ratios of 6 Gy, the McNamara RBE model on average does not predict much higher RBE values than 1.1 (see Fig. 3, first panel).

By analyzing volumes receiving at least 95% of the prescribed dose using cRBE and vRBE we estimated the average biological range extension of over 0.4 cm. This result is similar to the predictions of biological range with vRBE obtained by comparing the edges of high dose regions (i.e. range at 80% of the prescribed dose) in Giovannini et al. [34] and Grün et al. [21]. We also showed that incorporating the vRBE model, the range of D_RBE_ distributions in brain and skull base patients was extended on a similar level in both groups and was not correlated with the volume of the target exposed to high dose (more than 95% of prescribed dose). Minimizing the extension of the proton range and not exceeding dose limits in normal tissue is expected to be the factor limiting brain toxicity in H&N patients [35].

Monte Carlo simulation methods provide a reliable prediction of dose deposited in patients [22, 36]. Due to the wide availability of inexpensive GPU cards providing high computational performance, the MC calculation algorithms, including implementation of vRBE models can support clinical routine, helping to identify the regions potentially exposed to increased LET_d_ and/or D_RBE_. As our results show, the MC simulations indicate that real dose distribution can be more inhomogeneous than predicted by the analytical TPS, which results in lower mean dose in PTV using the same RBE model, which was also reported in Tseng et al. [37] and Ytre-Hauge et al. [38]. The RBE value depends on several variables and varies from patient to patient. It was shown that median RBE values may vary between patients by up to 15% [38].

We suggest that MC calculations of LET_d_ and variable RBE should support the treatment plan evaluation. Since including vRBE modeling in clinical treatment planning is still controversial, we believe that treatment planning optimization in the near future should include simultaneous optimization of LET_d_ and biological dose with cRBE in critical structures [39]. This will allow to reduce vRBE in OAR without substantial modification of biologically weighted dose computed with cRBE. Our results show that the region of the high dose computed with vRBE may expand significantly in comparison to cRBE. We should particularly consider these cases, when the PTV area is located near to the critical structures, which, exposed to high doses, can be affected by side effects after proton therapy. The short time needed for dose calculation in FRED enables vRBE evaluation in parallel to the clinical treatment planning workflow, while not affecting the treatment plan preparation time.

## Conclusions

As presented in this work, incorporating the vRBE approach would modify the D_RBE_ distribution, leading to an additional source of D_RBE_ and range uncertainty in treatment planning. We suggest that the treatment planning procedure should account for the uncertainty of the D_RBE_, particularly in high dose regions, predicted with the vRBE models. The biological range extension due to vRBE can be even larger than planned margins in PTV. Our approach of estimating the biological range extension is a suitable method for IMPT plans because, differently to the analysis of transverse profiles, is insensitive to dose inhomogeneities and radiation field directions.

## Supplementary Information

**Additional file 1: Table S1.** Patient database grouped by tumor type with information on prescribed dose, PTV volume and diagnosis. For the skull base patients the reported volume is from the first stage of treatment.

## Data Availability

The datasets generated during and/or analysed during the current study are available from the corresponding author on reasonable request.

## References

[1] Mendenhall NP, Malyapa RS, Su Z, Yeung D, Mendenhall WM, Li Z (2011). Proton therapy for head and neck cancer: rationale, potential indications, practical considerations, and current clinical evidence. Acta Oncol.

[2] Sreeraman R, Indelicato DJ (2014). Proton therapy for the treatment of children with CNS malignancies. CNS Oncol.

[3] Paganetti H (2016). Proton therapy physics.

[4] Paganetti H (2014). Relative biological effectiveness (RBE) values for proton beam therapy. Variations as a function of biological endpoint, dose, and linear energy transfer. Phys Med Biol.

[5] Lomax AJ (2019). Myths and realities of range uncertainty. Br J Radiol.

[6] Marshall TI, Chaudhary P, Michaelidesová A, Vachelová J, Davídková M, Vondráček V (2016). Investigating the implications of a variable RBE on proton dose fractionation across a clinical pencil beam scanned spread-out Bragg peak. Int J Radiat Oncol Biol Phys.

[7] Ilicic K, Combs SE, Schmid TE (2018). New insights in the relative radiobiological effectiveness of proton irradiation. Radiat Oncol.

[8] Sørensen BS, Pawelke J, Bauer J, Burnet NG, Dasu A, Høyer M (2021). Does the uncertainty in relative biological effectiveness affect patient treatment in proton therapy?. Radiother Oncol.

[9] Krishna GS, Srinivas V, Ayyangar KM, Reddy PY (2016). Comparative study of old and new versions of treatment planning system using dose volume histogram indices of clinical plans. J Med Phys.

[10] Bodensteiner D (2018). RayStation: External beam treatment planning system. Med Dosim.

[11] Perl J, Shin J, Schumann J, Faddegon B, Paganetti H (2012). TOPAS: an innovative proton Monte Carlo platform for research and clinical applications. Med Phys.

[12] Sarrut D, Bardiès M, Boussion N, Freud N, Jan S, Létang J-M (2014). A review of the use and potential of the GATE Monte Carlo simulation code for radiation therapy and dosimetry applications. Med Phys.

[13] Battistoni G, Cerutti F, Fassò A, Ferrari A, Muraro S, Ranft J, et al. The FLUKA code: description and benchmarking. In: AIP conference proceedings, AIP; 2007. p. 31–49.

[14] Schiavi A, Senzacqua M, Pioli S, Mairani A, Magro G, Molinelli S (2017). Fred: a GPU-accelerated fast-Monte Carlo code for rapid treatment plan recalculation in ion beam therapy. Phys Med Biol.

[15] Giantsoudi D, Schuemann J, Jia X, Dowdell S, Jiang S, Paganetti H (2015). Validation of a GPU-based Monte Carlo code (gPMC) for proton radiation therapy: clinical cases study. Phys Med Biol.

[16] Guterres Marmitt G, Pin A, Ng Wei Siang K, Janssens G, Souris K, Cohilis M, Langendijk JA, Both S, Knopf A, Meijers A (2020). Platform for automatic patient quality assurance via Monte Carlo simulations in proton therapy. Phys Med.

[17] Paganetti H (2017). Relating the proton relative biological effectiveness to tumor control and normal tissue complication probabilities assuming interpatient variability in α/β. Acta Oncol.

[18] Guan F, Peeler C, Bronk L, Geng C, Taleei R, Randeniya S (2015). Analysis of the track- and dose-averaged LET and LET spectra in proton therapy using the geant4 Monte Carlo code. Med Phys.

[19] Unkelbach J, Botas P, Giantsoudi D, Gorissen BL, Paganetti H (2016). Reoptimization of intensity modulated proton therapy plans based on linear energy transfer. Int J Radiat Oncol Biol Phys.

[20] Traneus E, Ödén J (2019). Introducing proton track-end objectives in intensity modulated proton therapy optimization to reduce linear energy transfer and relative biological effectiveness in critical structures. Int J Radiat Oncol Biol Phys.

[21] Grün R, Friedrich T, Krämer M, Zink K, Durante M, Engenhart-Cabillic R (2013). Physical and biological factors determining the effective proton range. Med Phys.

[22] Gajewski J, Garbacz M, Chang C-W, Czerska K, Durante M, Krah N, Krzempek K, Kopeć R, Lin L, Mojżeszek N, Patera V, Pawlik-Niedzwiecka M, Rinaldi I, Rydygier M, Pluta E, Scifoni E, Skrzypek A, Tommasino F, Schiavi A, Rucinski A (2020). Commissioning of GPU-accelerated Monte Carlo code Fred for clinical applications in proton therapy. Front Phys.

[23] Carabe A, Moteabbed M, Depauw N, Schuemann J, Paganetti H (2012). Range uncertainty in proton therapy due to variable biological effectiveness. Phys Med Biol.

[24] McNamara AL, Schuemann J, Paganetti H (2015). A phenomenological relative biological effectiveness (RBE) model for proton therapy based on all published in vitro cell survival data. Phys Med Biol.

[25] Scholz M, Kellerer AM, Kraft-Weyrather W, Kraft G (1997). Computation of cell survival in heavy ion beams for therapy. The model and its approximation. Radiat Environ Biophys.

[26] van Leeuwen CM, Oei AL, Crezee J, Bel A, Franken NAP, Stalpers LJA (2018). The alfa and beta of tumours: a review of parameters of the linear-quadratic model, derived from clinical radiotherapy studies. Radiat Oncol.

[27] Polster L, Schuemann J, Rinaldi I, Burigo L, McNamara AL, Stewart RD (2015). Extension of TOPAS for the simulation of proton radiation effects considering molecular and cellular endpoints. Phys Med Biol.

[28] Aditya Panchal, pyup.io bot, Gabriel Couture, gertsikkema, Nicolas Galler, Hideki_Nakamoto, David C Hall, Akihisa Wakita. dicompyler/dicompyler-core v0.5.5. 2019. https://doi.org/10.5281/zenodo.3236628.

[29] Wilcoxon F (1945). Individual comparisons by ranking methods. Biometrics Bulletin.

[30] McNamara AL, Willers H, Paganetti H (2020). Modelling variable proton relative biological effectiveness for treatment planning. Br J Radiol.

[31] Missiaggia M, Cartechini G, Scifoni E, Rovituso M, Tommasino F, Verroi E (2020). Microdosimetric measurements as a tool to assess potential in-field and out-of-field toxicity regions in proton therapy. Phys Med Biol.

[32] Tommasino F, Widesott L, Fracchiolla F, Lorentini S, Righetto R, Algranati C (2020). Clinical implementation in proton therapy of multi-field optimization by a hybrid method combining conventional PTV with robust optimization. Phys Med Biol.

[33] Yepes P, Adair A, Frank SJ, Grosshans DR, Liao Z, Liu A (2019). Fixed- versus variable-RBE computations for intensity modulated proton therapy. Adv Radiat Oncol.

[34] Giovannini G, Böhlen T, Cabal G, Bauer J, Tessonnier T, Frey K (2016). Variable RBE in proton therapy: comparison of different model predictions and their influence on clinical-like scenarios. Radiat Oncol.

[35] Lühr A, von Neubeck C, Krause M, Troost EGC (2018). Relative biological effectiveness in proton beam therapy—current knowledge and future challenges. Clin Transl Radiat Oncol.

[36] Testa M, Schümann J, Lu H-M, Shin J, Faddegon B, Perl J (2013). Experimental validation of the TOPAS Monte Carlo system for passive scattering proton therapy. Med Phys.

[37] Tseng YD, Maes SM, Kicska G, Sponsellor P, Traneus E, Wong T (2019). Comparative photon and proton dosimetry for patients with mediastinal lymphoma in the era of Monte Carlo treatment planning and variable relative biological effectiveness. Radiat Oncol.

[38] Ytre-Hauge KS, Fjæra LF, Rørvik E, Dahle TJ, Dale JE, Pilskog S (2020). Inter-patient variations in relative biological effectiveness for cranio-spinal irradiation with protons. Sci Rep.

[39] McMahon SJ, Paganetti H, Prise KM (2018). LET-weighted doses effectively reduce biological variability in proton radiotherapy planning. Phys Med Biol.

