# Supplementary material for: Quantification of biological range uncertainties in patients treated at the Krakow proton therapy centre
Source: Radiat Oncol. 2022 Mar 9;17:50. doi: 10.1186/s13014-022-02022-5 (PMC8905899; doi:10.1186/s13014-022-02022-5)
Supplement: Supplementary file 1 — Additional file 1: Table S1. Patient database grouped by tumor type with information on prescribed dose, PTV volume and diagnosis. For the skull base patients the reported volume is from the first stage of treatment. [file 13014_2022_2022_MOESM1_ESM.docx]

Table S1. Patient database grouped by tumor type with information on prescribed dose, PTV volume and diagnosis. For the skull base patients the reported volume is from the first stage of treatment.

| Brain patients | | | | Skull base patients | | | |
| --- | --- | --- | --- | --- | --- | --- | --- |
| Patient no | D_p_ [Gy(RBE)] | Vol. PTV [cm^3^] | Diag-nosis | Patient no | D_p_ [Gy(RBE)] | Vol. PTV [cm^3^] | Diag-nosis |
| 1 | 61.2 | 365.85 | GLI | 51 | 74.0 | 131.93 | CHS |
| 2 | 55.4 | 141.57 | GLI | 52 | 70.0 | 69.00 | CHS |
| 3 | 54.0 | 127.09 | GLI | 53 | 70.0 | 41.75 | CHS |
| 4 | 54.9 | 569.86 | GLI | 54 | 74.0 | 75.46 | CH |
| 5 | 54.0 | 266.29 | GLI | 55 | 74.0 | 127.38 | CHS |
| 6 | 54.6 | 433.63 | GLI | 56 | 74.0 | 266.59 | CHS |
| 7 | 54.0 | 734.17 | GLI | 57 | 70.0 | 165.43 | CHS |
| 8 | 60.0 | 391.45 | GLI | 58 | 74.0 | 252.19 | CH |
| 9 | 54.0 | 559.51 | GLI | 59 | 74.0 | 90.35 | CH |
| 10 | 54.0 | 591.41 | GLI | 60 | 70.0 | 134.52 | CHS |
| 11 | 60.0 | 567.48 | GLI | 61 | 74.0 | 172.75 | CH |
| 12 | 54.0 | 313.19 | GLI | 62 | 74.0 | 273.55 | CH |
| 13 | 54.0 | 729.47 | GLI | 63 | 74.0 | 99.71 | CH |
| 14 | 54.0 | 385.57 | GLI | 64 | 74.0 | 57.91 | CH |
| 15 | 54.0 | 366.28 | GLI | 65 | 74.0 | 277.89 | CH |
| 16 | 54.0 | 314.37 | GLI | 66 | 74.0 | 202.80 | CH |
| 17 | 54.0 | 476.69 | GLI | 67 | 74.0 | 88.16 | CHS |
| 18 | 54.0 | 1009.6 | GLI | 68 | 74.0 | 297.87 | CH |
| 19 | 54.0 | 527.16 | GLI | 69 | 70.0 | 22.39 | CHS |
| 20 | 54.0 | 823.05 | GLI | 70 | 74.0 | 63.36 | CH |
| 21 | 54.0 | 291.47 | GLI | 71 | 74.0 | 117.65 | CH |
| 22 | 50.4 | 155.68 | GLI | 72 | 74.0 | 156.86 | CH |
| 23 | 54.0 | 446.60 | GLI | 73 | 74.0 | 367.93 | CH |
| 24 | 54.0 | 403.84 | GLI | 74 | 74.0 | 116.02 | CH |
| 25 | 36.0 | 70.53 | GLI | 75 | 74.0 | 188.64 | CH |
| 26 | 60.0 | 436.96 | GLI | 76 | 70.0 | 125.31 | CHS |
| 27 | 60.0 | 514.18 | GLI | 77 | 70.0 | 386.11 | CHS |
| 28 | 54.0 | 388.07 | GLI | 78 | 74.0 | 133.28 | CH |
| 29 | 60.0 | 58.95 | GLI | 79 | 70.0 | 112.51 | CHS |
| 30 | 54.0 | 160.88 | GLI | 80 | 74.0 | 440.52 | CH |
| 31 | 54.0 | 432.83 | GLI | 81 | 70.0 | 105.42 | CHS |
| 32 | 54.0 | 416.62 | GLI | 82 | 70.6 | 64.22 | CH |
| 33 | 54.0 | 223.62 | GLI | 83 | 74.0 | 141.26 | CH |
| 34 | 50.0 | 128.74 | GLI | 84 | 70.0 | 278.52 | CH |
| 35 | 54.0 | 187.50 | GLI | 85 | 74.0 | 245.87 | CH |
| 36 | 54.0 | 162.33 | GLI | 86 | 70.0 | 182.17 | CHS |
| 37 | 45.0 | 199.19 | GLI | 87 | 74.0 | 77.00 | CH |
| 38 | 54.0 | 265.73 | GLI | 88 | 74.9 | 92.59 | CH |
| 39 | 54.0 | 213.22 | GLI | 89 | 70.0 | 85.82 | CHS |
| 40 | 54.0 | 274.36 | GLI | 90 | 74.0 | 95.83 | CH |
| 41 | 45.0 | 235.28 | GLI | 91 | 74.0 | 87.23 | CHS |
| 42 | 54.0 | 170.84 | GLI | 92 | 70.0 | 147.28 | CHS |
| 43 | 52.2 | 295.16 | GLI | 93 | 74.0 | 73.63 | CH |
| 44 | 52.2 | 185.54 | GLI | 94 | 74.0 | 93.15 | CH |
| 45 | 54.0 | 151.21 | GLI | 95 | 74.0 | 122.84 | CHS |
| 46 | 54.0 | 329.92 | GLI |  |  |  |  |
| 47 | 36.0 | 75.22 | GLI |  |  |  |  |
| 48 | 54.0 | 136.61 | GLI |  |  |  |  |
| 49 | 54.0 | 67.60 | GLI |  |  |  |  |
| 50 | 54.0 | 73.50 | GLI |  |  |  |  |

* D_p_ - prescribed dose, GLI - glioma, CH - chordoma, CHS - chondrosarcoma.
